# A case report of endorectal displacement of a right ureteral stent following radiochemotherapy and Bevacizumab

**DOI:** 10.1186/s12894-019-0566-1

**Published:** 2019-12-09

**Authors:** Alessio Tognarelli, Lorenzo Faggioni, Francesca Manassero, Angiolo Gadducci, Cesare Selli

**Affiliations:** 1 Department of Translational Research and New Technologies in Medicine and Surgery, Sections of Urology, University of Pisa, via Paradisa 2, 56126 Pisa, Italy; 2 Diagnostic and Interventional Radiology, University of Pisa, Pisa, Italy; 3 Department of Clinical and Experimental Medicine, Section of Gynecologic Oncology, University of Pisa, Pisa, Italy

**Keywords:** Ureteral stent complications, Angiogenesis inhibitors, CT scan, Urinary fistula

## Abstract

**Background:**

The angiogenesis inhibitor monoclonal antibody Bevacizumab is presently the standard treatment for numerous neoplasms but particular toxicities are emerging, such as hypertension, haemorrhage, thromboembolism, gastrointestinal perforation, fistulae, and delayed wound healing. The addition of Bevacizumab to radio and chemotherapy has improved the overall survival rate in patients with metastatic, persistent or recurrent cervical carcinoma. However an increased risk of enteric or urinary fistula formation has been documented, related to hypoxia which is induced by the inhibition of angiogenesis. Moreover, previous pelvic surgery, repeated ureteral stenting and radiation are additional risk factors.

**Case presentation:**

We describe the remarkable case of a right ureteral stent displacement inside the rectum lumen in a patient treated with Bevacizumab for pelvic recurrence of cervical cancer. The patient was referred to our Urology Department with urinary sepsis and bilateral hydronephrosis. Right ureteral stent substitution was planned; at cystoscopy the distal loop of the stent was not visualized inside the bladder. The presence of the distal loop of the right ureteral inside the rectum was clearly demonstrated with a CT scan.

**Conclusions:**

Since Bevacizumab is increasingly used in the treatment of gynaecological neoplasms and indwelling ureteral stents are often required to treat or prevent ureteral compressions, similar cases are likely to be diagnosed and this complication should be considered in the management of advanced pelvic cancers.

## Background

The association of monoclonal antibodies causing angiogenesis inhibition, like Bevacizumab, to radio and chemotherapy is known to increase the incidence of fistulae [1]. In particular, the final analysis of a large randomized prospective trial on the use of Bevacizumab in women with advanced cancer of the cervix, demonstrated an advantage in the overall survival rate compared to chemotherapy alone (16.8 vs 13.3 months) but also an increased risk of fistula formation (15% vs 1%) [2]. Of note, all the women with fistulae had previously been irradiated and their history of smoking was an associated risk factor. The fistulae involved the genitourinary tract in 7% of cases and the gastrointestinal [tract] in 8%.

Bevacizumab is, at present, the standard treatment for numerous neoplasms, and particular toxicities are emerging which may cause major morbidity and even mortality [3]. Ischemia and an impaired function of nitrous oxide, prostacyclins and platelets due to VEGF inhibition are the likely causes of increased fistula formation. Additional risk factors for fistulae involving the urinary tract are represented by previous pelvic surgery, repeated ureteral stenting and mostly [do you mean ‘above all’/ ‘most of all’?] radiation, due to its additional toxicity on microvasculature. Moreover, the positioning of ureteral stents is often required in advanced pelvic cancer to prevent or treat hydroureteronephrosis.

Herein, we report the case of a female patient with a diagnosis of cervical cancer recurrence treated with Bevacizumab, who was referred to our Urology Unit for hydronephrosis and sepsis; the patient had an indwelling right ureteral stent, whose distal loop was found dislocated in the rectal lumen at CT scan.

## Case presentation

A 40-year-old woman was referred to our Urology Department with a diagnosis of urinary sepsis and bilateral hydronephrosis; radical hysterectomy, bilateral salpingectomy with ovarian preservation as well as pelvic and para-aortic lymphadenectomy for squamous cell carcinoma of the cervix had been performed 8 years earlier. The patient received adjuvant concurrent cisplatin-based chemo radiotherapy up to a total dose of 50.4 Gy; next she underwent periodical surveillance examinations which resulted negative for long term. Twenty months earlier a CT scan revealed a right-sided pelvic recurrence involving the right ureter with concurrent hydronephrosis; treatment of the recurrence required 3 further cycles of Cisplatin, Paclitaxel and Bevacizumab, obtaining a partial response at 18F-FDG PET/CT, followed by additional cycles of Bevacizumab every 3 weeks as maintenance treatment. A right ureteral stent was placed with the retrograde cystoscopic approach at the time of recurrence diagnosis to treat the associated hydronephrosis and had already been substituted twice using the same approach without problems employing hydrophilic long-permanence stents.

At time of the admission, a urinary tract infection sustained by Enterococcus was under treatment with Linezolid; abdominal sonography revealed bilateral hydronephrosis, with the presence of the curled upper extremity of the stent inside the right kidney collecting system, but the lower extremity was not detected in the bladder. Substitution of the right ureteral stent was planned to treat the sepsis.

At cystoscopy the distal end of the stent was not visible inside the bladder, while a fistula orifice covered with fibrin was evident on the right side of the bladder trigone, so the planned procedure was suspended.

**Fig. 1:**
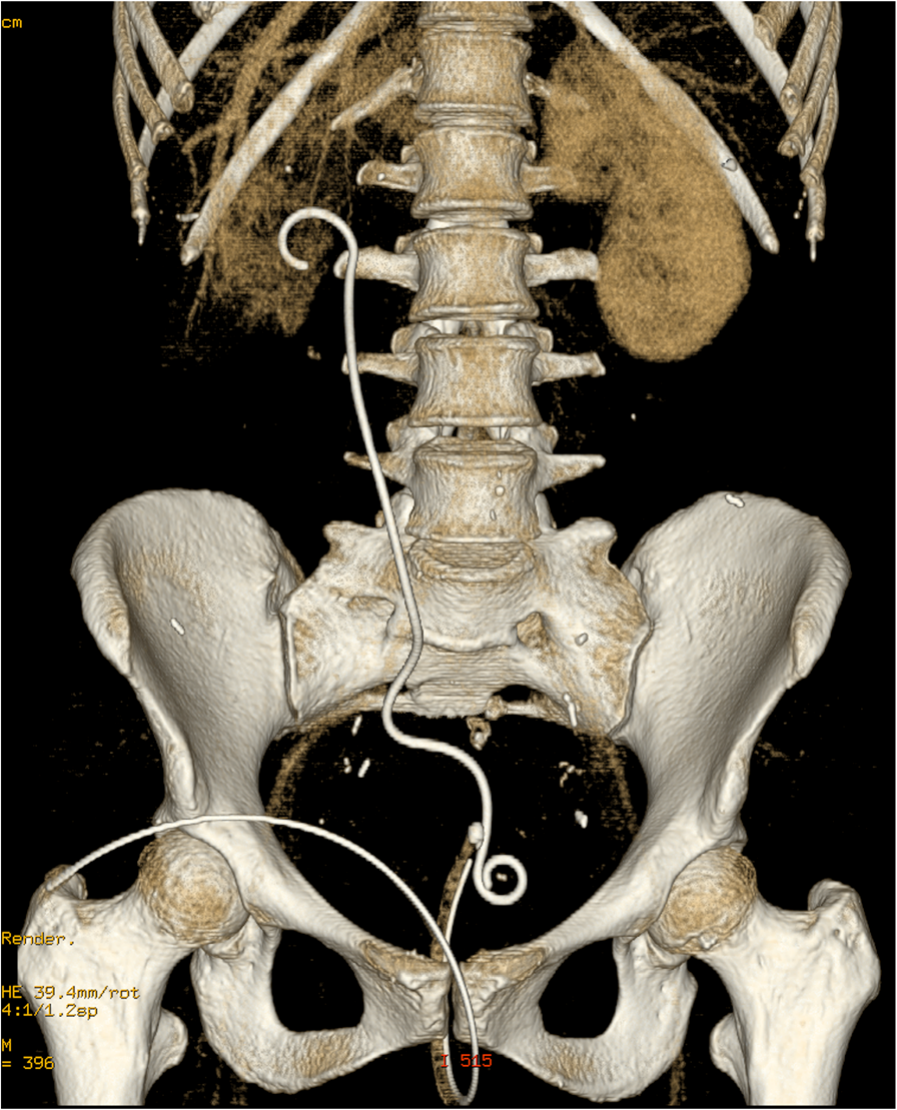
3D rendering of the CT scan showing a cross-over course of the right ureteral stent at the level of mid-sacrum, while the stent distal coil is placed on the left side of the midline, defined by the presence of the bladder catheter

**Fig. 2:**
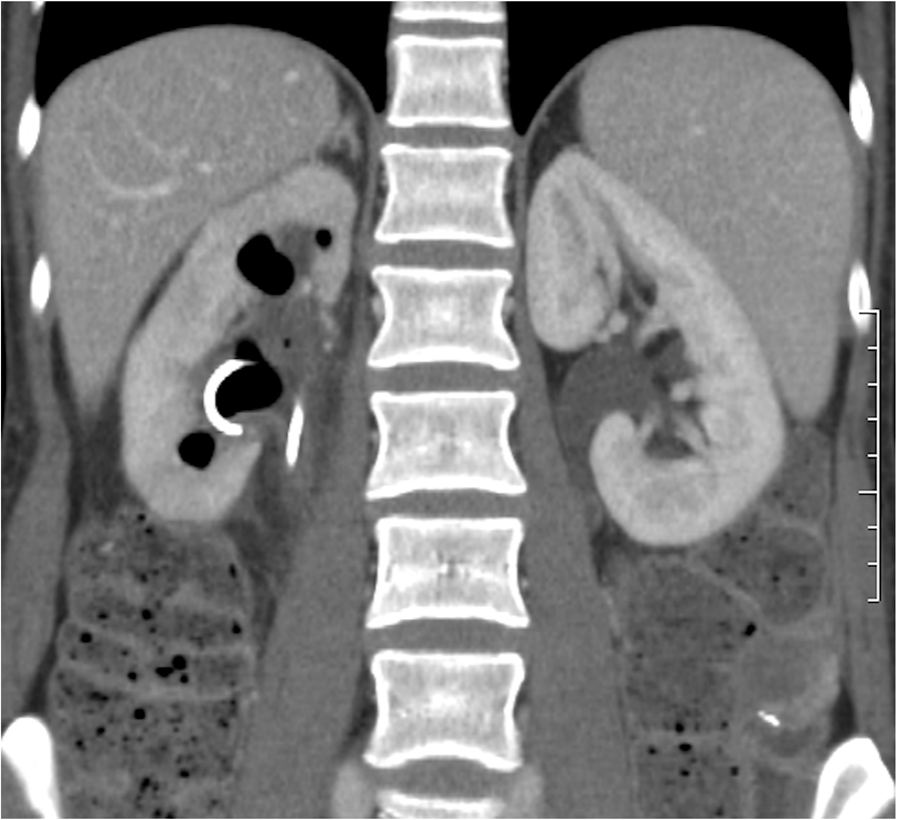
Coronal section of the CT scan in a late-venous phase revealing presence of gas inside the right pyelo-calyceal system and the upper coil of the double J stent

**Fig. 3:**
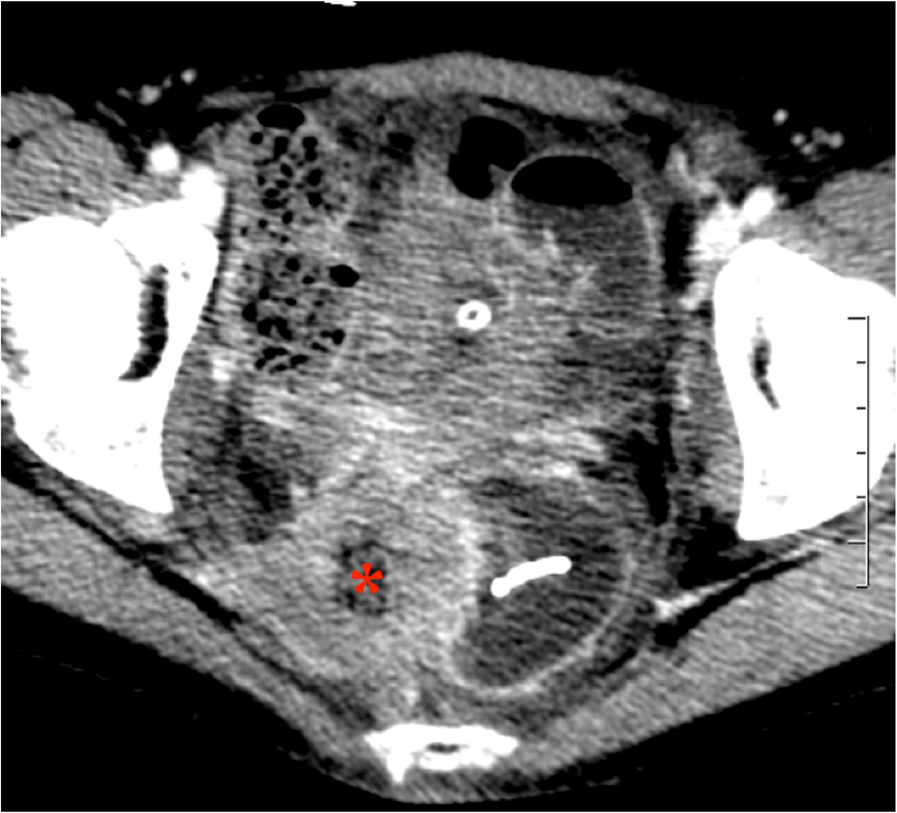
Axial section of the CT scan in a venous phase demonstrating a right sided pelvic tumor recurrence with a partial central necrosis. The distal coil of the stent inside the rectal lumen and the bladder catheter are also visible

A 64-detector row multiphase CT examination of the abdomen and pelvis was performed, showing a cross-over course of the ureteral stent from the right side to the left at the level of the sacrum, which was more evident with 3D rendering (Fig. 1), presence of gas inside the right pyelocalyceal system, along the side of the upper coil of the stent (Fig. 2) with displacement of the distal third of the stent and its lower loop inside the rectum, and right-sided pelvic tumor recurrence (Fig. 3). A delayed scan revealed the presence of iodinated contrast material inside the rectal lumen, consistent with urinary fistulization. Subsequent treatment consisted in bilateral percutaneous nephrostomy in the prone position to relieve hydronephrosis and digital extraction of the stent from the rectal ampulla; an indwelling bladder catheter was placed. Two days later a left excluding colostomy was performed.

## Discussion and conclusions

The present case represents, to the best of our knowledge, the first documentation with imaging techniques of the migration of a double J ureteral stent to the rectal lumen through tumor necrosis after treatment with Bevacizumab.

In the only other case of double J ureteral stent displacement inside the rectum that we are aware of, the diagnosis was made by means of colonoscopy of a woman who had been treated with radiotherapy and previous colostomy for pelvic recurrence of cervical cancer and presented with rectal bleeding [4]. Bilateral ureteral stents had been positioned 4 months earlier to relieve hydronephrosis.

Since indwelling ureteral stents are increasingly used in the management of patients with advanced gynaecological cancers where the use of Bevacizumab has become standard therapy, it is not unlikely that similar cases will be diagnosed in the future. As in our case, conventional radiology used during standard endourologic procedures is of limited help in identifying the endorectal displacement of the distal end of a double J ureteral stent, at least in the antero-posterior view. Similarly, sonography may fail to identify the absence of the distal extremity of the stent inside the bladder when it is empty or when a urinary catheter is in place. On the contrary, a CT scan provides an optimal demonstration of stent displacement and associated pelvic lesions, and gives important clues for further treatment planning. More generally, a multiphase contrast-enhanced CT allows a comprehensive assessment of the urinary tract, presence and extent of disease recurrence, and potential treatment-related complications (such as urinary fistulae) with a fast, one-stop examination. As known, the now widespread availability of CT scanners with 64 or more detector rows allows a routine acquisition of images with submillimeter spatial resolution and voxel isotropy, enabling high quality 2D and 3D image processing that yields an accurate, realistic depiction of all relevant structures under investigation [5]. Furthermore, the adoption of iterative reconstruction algorithms on modern multidetector CT equipment can significantly reduce patient exposure to ionizing radiation while maintaining the diagnostic image quality [6]. It is also worth mentioning that, in addition to mere morphological data, more advanced technological implementations of modern multidetector CT imaging (including CT perfusion and dual energy CT) and emerging applications such as CT texture analysis in oncology can provide functional information about cancer status that could help predict an early response to antiangiogenic agents and the overall patient outcome [7]. In light of this and given the increased use of Bevacizumab for the treatment of several neoplastic diseases, both clinicians and radiologists should be aware of the potential complications related to Bevacizumab treatment and so be prepared to harness the great potential of a multidetector CT to reach an accurate diagnosis, therefore possibly leading to better patient care.

Fistulization between the lower urinary tract and the rectum in the present case was, in our opinion, related to shrinkage and necrosis of the pelvic recurrence caused by angiogenesis inhibitors, which also increased wall fragility of the pelvic organs.

In order to mitigate the risk of fistulization the Urologists should try to monitor and treat concomitant urinary tract infections, use hydrophilic stents of low gauge and avoid traumatic manoeuvres during stent substitution. Advances in stent materials, such as antibacterial coating, are likely to reduce the risk of fistulization [8], while the use of metallic stents in malignant ureteral obstruction has proven to be cost-effective [9], but their increased stiffness potentially increases the risk of tissue damage.

## Data Availability

All data generated or analysed during this study are included in this published article.

## Abbreviations

^18^F-FDG PET/CT: fluorodeoxyglucose positron emission tomography / CT computed tomography
CT: Computed Tomography
Gy: Gray
VEGF: Vascular Endothelial Growth Factor
